# Reviewer acknowledgement 2014

**DOI:** 10.1186/s12959-015-0041-5

**Published:** 2015-02-24

**Authors:** Yukio Ozaki, Hugo ten Cate

**Affiliations:** University of Yamanashi, Yamanashi, Japan; Academic Hospital Maastricht, Maastricht, The Netherlands

## Abstract

The Editors of *Thrombosis Journal* would like to thank all of our reviewers who have contributed to the journal in Volume 12 (2014) and whose valuable support is fundamental to its success.

**Abdulkareem Almomen**

Saudi Arabia

**Khalid Alsaleh**

Saudi Arabia

**Tamam Bakchoul**

Germany

**Rupert Bauersachs**

Germany

**Isabell Bernlochner**

Germany

**Jan Beyer-Westendorf**

Germany

**Jean-Claude Bordet**

France

**Annemieke Bouman**

Netherlands

**Henri Bounameaux**

Switzerland

**Sigrid Brækkan**

Norway

**Luca Cantarini**

Italy

**Jennifer Curnow**

Australia

**Kesheng Dai**

China

**Anthonius De Boer**

Netherlands

**Edwin de Vrij**

Netherlands

**Mario Del Rosso**

Italy

**Annemieke Dhondt**

Belgium

**Matteo Nicola Dario Di Minno**

Italy

**Robert Di Domenico**

United States of America

**Paul Dobesh**

United States of America

**Pieter Eijgenraam**

Netherlands

**Bengt I Eriksson**

Sweden

**Petra Erkens**

Netherlands

**John Fanikos**

United States of America

**Satoshi Fujii**

Japan

**Esteban Gandara**

Canada

**Hadi Goubran**

Canada

**Sylvia Haas**

Germany

**Adrian V Hernandez**

Netherlands

**Soudabeh Hossini**

Iran

**Karim Ibrahim**

Germany

**Masahiro Ieko**

Japan

**Zafar Iqbal**

Pakistan

**Takayuki Iwaki**

Japan

**Manuela Joore**

Netherlands

**Joanne Joseph**

Australia

**Makoto Kaneko**

Japan

**Renate Kaulitz**

Germany

**Isao Kitajima**

Japan

**Marie-Claire Kleinegris**

Netherlands

**Ralf Knöfler**

Germany

**Stavros Konstantinides**

Germany

**Mirjana Kovac**

Serbia

**Legese Kumsa**

Ethiopia

**Riitta Lassila**

Finland

**Marcel Levi**

Netherlands

**Tomas Lindahl**

Sweden

**Elena Lipets**

Russian Federation

**Marina Marchetti**

Italy

**Karina Meijer**

Netherlands

**Pier Luigi Meroni**

Italy

**Marco Moia**

Italy

**Manuel Monreal**

Spain

**Tomoaki Morioka**

Japan

**Eriko Morishita**

Japan

**Caroline Nilsson**

Sweden

**Michael Nurmohamed**

Netherlands

**Rene van Oerle**

Netherlands

**Tsukasa Ohmori**

Japan

**Martin Olivieri**

Germany

**Mikhail Ovanesov**

United States of America

**Flora Peyvandi**

Italy

**Olivia Phung**

United States of America

**Ron Pisters**

Netherlands

**Paolo Prandoni**

Italy

**Hanne Berg Ravn**

Denmark

**Dick Rijken**

Netherlands

**Eduardo Rocha**

Brazil

**Christian Ruff**

United States of America

**Ulrich Sachs**

Germany

**Masato Sakon**

Japan

**Michel Meyer Samama**

France

**Katrin Schaefer**

Germany

**Craig Seaman**

United States of America

**Keiko Shinozawa**

Japan

**Harkirat Singh**

United States of America

**Marijn Speeckaert**

Belgium

**Yuko Suzuki**

Japan

**Junki Takamatsu**

Japan

**Arina ten Cate-Hoek**

Netherlands

**Hiroko Tsuda**

Japan

**Anetta Undas**

Poland

**Felix van der Meer**

Netherlands

**Hideo Wada**

Japan

**Jeanine Walenga**

United States of America

**Jintao Wang**

United States of America

**Jun Wang**

China

**Xuefeng Wang**

China

**Theodore Warkentin**

Canada

**Kristien Winckers**

Netherlands

**Shinsuke Yasuda**

Japan

